# Corrosion Behavior of Reinforcing Steel Undergoing Stray Current and Anodic Polarization

**DOI:** 10.3390/ma14020261

**Published:** 2021-01-07

**Authors:** Zhipei Chen, Dessi Koleva

**Affiliations:** Faculty of Civil Engineering and Geosciences, Department 3MD, Section of Materials and Environment, Delft University of Technology, Stevinweg 1, 2628 CN Delft, The Netherlands; d.a.koleva@tudelft.nl

**Keywords:** stray current, anodic polarization, corrosion, steel, mortar

## Abstract

Different concrete structures (viaducts, bridges, or tunnels) in the neighborhoods of railways may be subject to the stray current leaking from the rails. In these cases, the reinforcing rebars embedded in concrete act as conductors, “pick up” the stray current, and can corrode. For simulating the stray current-induced corrosion of metals, most researchers just supplied anodic polarization on samples. However, stray current induces both cathodic polarization and anodic polarization. This work experimentally justifies the different effects of stray current and anodic polarization on reinforcing steel embedded in mortar. A comparison between stray current and anodic polarization effects on the corrosion behavior of embedded steel is performed for both fresh (24 hour-cured) and hardened matrix (28 day-cured) in chloride-free (Cl-free) and chloride-containing (Cl-containing) environments. It is found that in all studied conditions, anodic polarization leads to a significantly different electrochemical performance of the steel rebar compared to the stray current. Hence, anodic polarization cannot reflect all the effects of stray current, and therefore, it has limited significance for simulating stray current. It is also clarified that the curing regimes and starting time of the stray current play significant roles in the formation of a corrosion product layer on the steel surface.

## 1. Introduction

**Figure 1:**
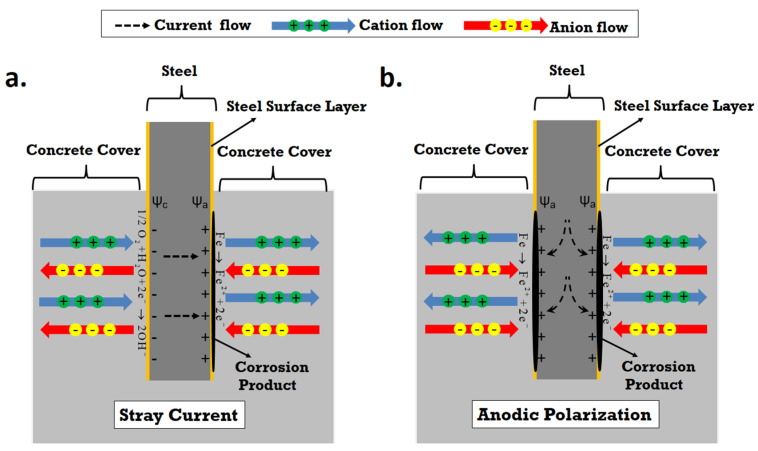
Ion migration in reinforced concrete undergoing: (**a**) Stray current; (**b**) Anodic polarization.

Currents flowing along paths not being elements of a purpose-built electric circuit are called stray currents [1,2]. Different concrete structures (viaducts, bridges, or tunnels) in the neighborhoods of railways may be subject to the stray current leaking from the rails. In these cases, the concrete pore solution acts as an electrolyte, and the reinforcing rebars embedded in concrete act as conductors, which can “pick up” the stray current and can corrode. The mechanism of stray current induced-corrosion of steel in concrete can be seen in Figure 1a. At the location where the stray current “enters” the steel, cathodic reaction occurs (predominantly oxygen reduction, in the high pH environment of pore solution of concrete matrix):

(1) $${1/2O}_{2}{+H}_{2}{O+2e}^{-}\to {2\mathrm{OH}}^{-}$$

At the point where the stray current flows out from the steel into the external environment (e.g., concrete), anodic reaction takes place (i.e., steel corrosion):

(2) $$\mathrm{Fe}\to {\mathrm{Fe}}^{2+}{+2e}^{-}$$

To study the effects of stray current on the corrosion behavior of steel in concrete, various approaches have been presented in various research papers [3].

However, as reported in [2,3], most of the investigations actually reported the results on anodic polarization rather than stray current-induced corrosion of steel in reinforced concrete specimens.

Although stray current leads to the anodic locations on a steel surface, meaning that the degradation itself is linked to anodic currents and oxidation, the influence of stray current is not just anodic polarization. As presented in Figure 1a, the stray current effect is composed of both anodic polarization and cathodic polarization on a steel surface. Hence, stray current and its effects are more complex than only anodic polarization. Except for exerting effects on the metallic conductor (steel specifically), stray current will also trigger ion migration in a cement-based matrix. Ion migration in an electrolyte is an ion transport mechanism that can only occur in an electrical field. As shown in Figure 1, cations would migrate in the direction of the current, but anions (e.g., Cl^−^) migrate in the opposite direction. Ion migration (or its co-existence with diffusion, capillary suction, etc.) in cement-based materials is through the connected pores [4]. Thus, the transport process is related to the porosity and pore network connectivity of the bulk, which is also determined by the age/maturity of a concrete matrix.

Fresh concrete at a very early age is considered as a viscoelastic material [5]. From mixing until the initial setting time, cement hydration takes place and makes the concrete harden. During this period, ion migration, due to the electrical field, would additionally influence the cement hydration process and product layer formation on the steel surface. Specifically, current flowing in the fresh cement matrix with high porosity and permeability can easily lead to enhanced water and ion transport due to accelerated ion migration. Consequently, cement hydration would be enhanced, faster development of the cementitious microstructure would be at hand, and the more rapid stabilization of the pore solution and hydration products would occur at the steel–mortar interface. In other words, the curing regimes and starting time of the stray current (i.e., the stray current supply starts at an early age or later age) may play significant roles in the formation of a corrosion product layer on the steel surface. However, this aspect has not been investigated before.

One of the aims of this work is to justify the different effects of stray current and anodic polarization on reinforcing steel embedded in “fresh” (24 hour-cured) and “matured” mortar (28 day-cured). Stray current and anodic polarization are both applied and monitored in identical reinforced mortar specimens. The specimens are cured in a fog room (98% RH, 20 °C) for only 24 hours (24h) to produce “fresh” bulk matrix or the standard 28 days (28d) for hardened matrix. For a period of 243 days of conditioning, a comparison between stray current and anodic polarization effects on the corrosion behavior of embedded steel is performed for both fresh (24h-cured) and hardened matrix (28d-cured) in a Cl-free and Cl-containing environment. It is found that in all conditions, anodic polarization leads to the significantly different electrochemical performance of the steel rebar, compared to stray current. Hence, anodic polarization cannot reflect all the effects of stray current, and therefore, it has limited significance for simulating stray current. It is also clarified that the curing regimes and starting time of stray current (i.e., the stray current supply starts at age of 24h or 28d) play significant roles in the formation of a corrosion product layer on the steel surface.

## 2. Materials and Methods

### 2.1. Materials and Specimen Preparation

**Figure 2:**
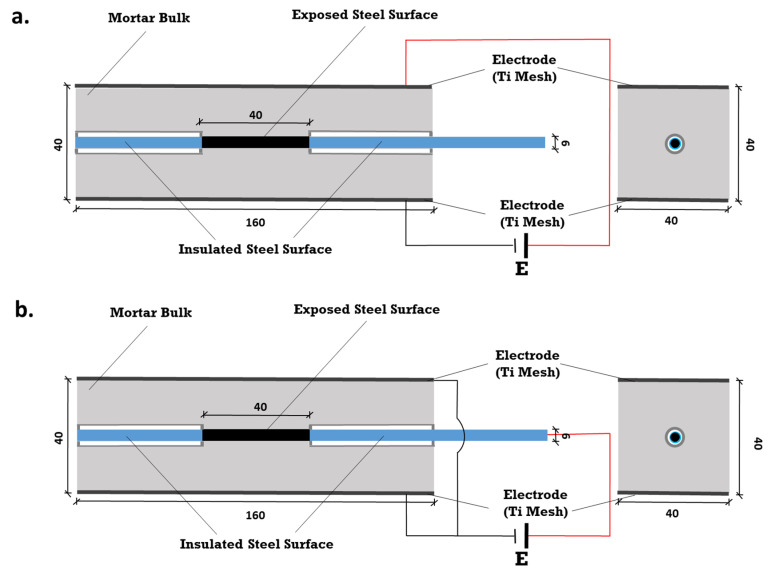
Experimental set-up for current supply and position of electrodes: (**a**) Stray current; (**b**) Anodic polarization.

Stray current and anodic polarization were applied on reinforced mortar prisms (of 40 × 40 × 160 mm^3^). The specimens were cast from Ordinary Portland Cement (OPC)—CEM I 42.5 N (ENCI, Maastricht, The Netherlands) and normed sand. The water-to-cement (W/C) ratio was 0.5; the cement-to-sand (C/S) ratio was 1:3. Construction steel (rebar) FeB500HKN (d = 6 mm) with an exposed length of 40 mm (with an exposed steel surface area of 7.54 cm^2^) was centrally embedded in the mortar prisms. The schematics of the specimens’ geometry is depicted in Figure 2.

Prior to casting, the steel rebars were cleaned electrochemically by the cathodic current of 100 A/m^2^, where the steel rebar was the cathode, and stainless steel was the anode. This process was performed in a solution of 75 g NaOH, 25 g Na_2_SO_4_, and 75 g Na_2_CO_3_ (reagent water to make 1000 mL), according to ASTM G-1 [6]. After that, the 2 ends of the rebar were covered by a heat-shrinkable tube. This aimed to avoid or minimize crevice corrosion and confine the effect of the experimental conditions to identical geometry and the exposed steel surface.

The two cast-in MMO (Mixed Metal Oxide) Ti electrodes (MMO Ti mesh, 40 × 160 mm^2^) served as terminals for anodic polarization and/or stray current application. When anodic polarization and/or stray current supplies were interrupted (min 24 hours before electrochemical tests), the two Ti electrodes (connected with each other) served as a counter electrode in a general 3-electrode system. In this system, the working electrode was rebar, and the reference electrode was an external Saturated Calomel Electrode (SCE). 

The stray current and anodic polarization level was set at 0.3 mA/cm^2^. The current density was calculated according to the exposed steel surface area. This current density was chosen according to a hypothetic 10% weight loss of steel rebar, as analytically calculated via Faraday’s law, for a period of 28 days in the relevant experimental conditions:

(3) $$i=ZFr\rho \eta_{s}/2At$$

where *t* is duration of corrosion (28 days = 2,419,200 s), *Z* is the valence of the iron ions (*Z* = 2), *F* is Faraday’s constant (96,500 As), *r* is the radius of the corroded bar (0.3 cm), *ρ* is the iron density (*ρ* = 7.87 g/cm^3^), *η_s_* is the mass loss ratio (10%), *A* is the atomic mass of iron (*A* = 56 g), and *i* is the current density (A/cm^2^). Based on this calculation, the anodic polarization level is 0.174 mA/cm^2^. Considering the fact that the supplied anodic current may be partially limited if any resistive components in the circuit would arise within the mortar, the current level was increased to 0.3 mA/cm^2^.

### 2.2. Curing and Conditioning

The relevant curing, conditioning regimes, and specimens designation are shown in Table 1. After casting, all specimens were cured in a fog room (98% RH, 20 °C) for 24 hours (24h) or 28 days (28d) until demolding. Next, the specimens were lab-conditioned (lab air). The specimens were submerged in water (Cl-free) or 5% NaCl solution (Cl-containing), with 2/3rd of height.

### 2.3. Experimental Methods

Electrochemical measurements were performed at Open Circuit Potential (OCP), using an SCE as a reference electrode (the counter electrode was the MMO Ti mesh). The OCP values were recorded at each time interval prior to electrochemical measurements.

In general, the OCP evolution provides information for transitions from passive to active state, and vice versa. For steel embedded in a cement-based material, a threshold value of −200 ± 70 mV (vs. SCE) [7] has been accepted, i.e., more anodic OCP values would reflect a passive state, whereas more cathodic values are linked to an active (corroding) state [8]. However, OCP only provides an indication of the corrosion state rather than giving quantitative information, e.g., corrosion rate. Hence, more cathodic OCP values would not always be related to increased corrosion rates. The OCP response of the steel can be affected by different factors, such as relative humidity, oxygen availability, and the resistance of the layers. For instance, limited oxygen availability (as in submerged conditions) can be reflected in a more cathodic OCP value. Hence, the interpretation of OCP values in such conditions would be more complex, and cathodic OCP would not indicate enhanced corrosion activity.

Linear Polarization Resistance (LPR) was conducted in the range of ± 20 mV (vs. OCP) at the scan rate of 0.1 mV/s (= 6 mV/min). It is found that the sweep rates of 2.5–10 mV/min give reliable results (suitability obtained by comparison to gravimetric losses, as reported in Ref. [9]). This range of scan rate makes sure the R_p_ achieves a constant value (i.e., the stationary value), because within this scan rate, the recorded i (vs. E) is already constant after the attenuation of varying current (this process is controlled by the attenuation rate, which is governed by the time constant of the working electrode) [9,10].

This method allows the determination of polarization resistance (R_p_). The R_p_ values can be used for calculating the corrosion current according to the Stern–Geary equation: i_corr_ = B/R_p_ [11]. As R_p_ is inversely proportional to the corrosion current, the quantification of corrosion resistance can be performed by comparing R_p_ values, as used and discussed in this work.

Electrochemical Impedance Spectroscopy (EIS) was performed in the frequency range of 50 kHz–10 mHz by superimposing an AC perturbation voltage of 10 mV (rms). As a non-destructive electrochemical technique, EIS provides both qualitative and quantitative information of steel reinforcement and bulk matrix. The high-frequency (HF) range (i.e., MHz to approximately 10 kHz) offers information for the contribution of the bulk matrix (solid and pore network). The high to middle frequency range (10 kHz to 1 kHz) reflects the contribution of the pore network and steel–mortar interface, while the middle (MF) to low frequency (LF) range (<1 kHz to 10 mHz) corresponds to the electrochemical performance of the steel [12].

For the 24h-cured specimens, both LPR and EIS tests were performed at the age of 3, 7, 14, 28, 56, 141, and 215 days. For the 28d-cured cases, LPR and EIS tests were conducted at the age of 28 (after 1d conditioning), 35 (after 7d conditioning), 42 (after 14d conditioning), 56 (after 28d conditioning), 169 (after 141d conditioning), and 243 (after 215d conditioning) days. In other words, the time periods of testing and conditioning for both 24h-cured and 28d-cured specimens were identical, but the hydration age of the specimens at the specific time interval varied, reflecting the 24h and 28d curing.

For specimens undergoing stray current or anodic polarization, a 24-h de-polarization (potential decay) was performed prior to any further testing. The experimental protocol and the sequence of tests are meant to verify the following: (1) if the stray current indeed flows into the steel; (2) if 24-hour potential decay is sufficient to result in stability of the electrochemical state of steel (i.e., if a stable OCP was achieved), so that electrochemical tests can follow after the decay. During the decay and within electrochemical tests, the specimens were immersed fully in the relevant medium (in water or 5% NaCl). The used equipment for electrochemical tests in this work was Metrohm Autolab (Potentiostat PGSTAT302N), combined with an FRA2 module.

## 3. Results and Discussion

### 3.1. OCP and R_p_ Evolution

**Figure 3:**
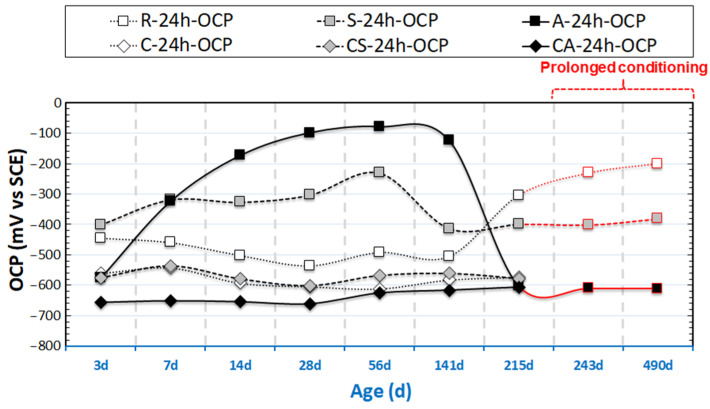
Open Circuit Potential (OCP) evolution of 24h-cured samples (R-24h: Reference; S-24h: Stray Current; A-24h: Anodic Polarization; C-24h: NaCl medium; CS-24h: NaCl + Stray Current; CA-24h: NaCl + Anodic Polarization).

**Figure 4:**
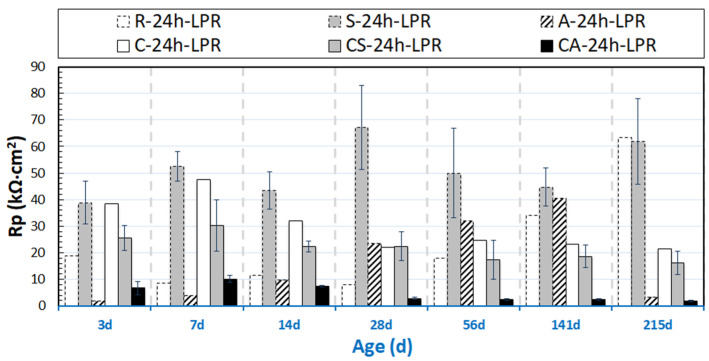
R_p_ evolution of 24h-cured samples (R-24h: Reference; S-24h: Stray Current; A-24h: Anodic Polarization; C-24h: NaCl medium; CS-24h: NaCl + Stray Current; CA-24h: NaCl + Anodic Polarization).

**Figure 5:**
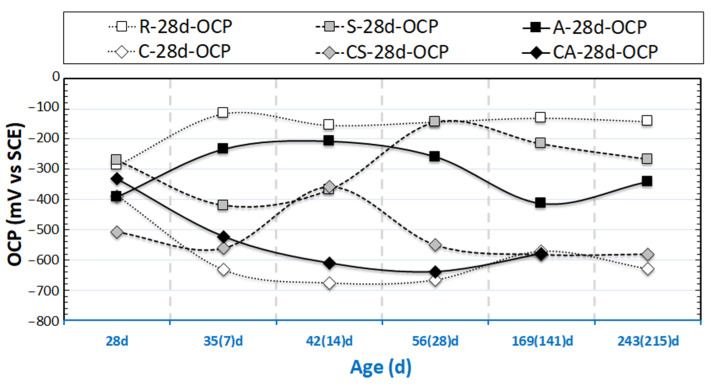
OCP evolution of 28d-cured samples (R-28d: Reference; S-28d: Stray Current; A-28d: Anodic Polarization; C-28d: NaCl medium; CS-28d: NaCl + Stray Current; CA-28d: NaCl + Anodic Polarization); The numbers in brackets denote the conditioning duration.

**Figure 6:**
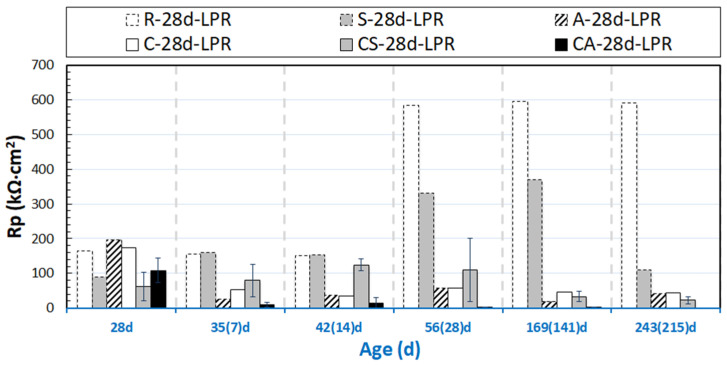
R_p_ evolution of 28d-cured samples (R-28d: Reference; S-28d: Stray Current; A-28d: Anodic Polarization; C-28d: NaCl medium; CS-28d: NaCl + Stray Current; CA-28d: NaCl + Anodic Polarization); The numbers in brackets denote the conditioning duration.

The evolution of OCP and R_p_ values (derived from LPR) for the group of 24h-cured specimens are presented in Figure 3 and Figure 4. Figure 5 and Figure 6 depict the OCP and LPR records of the 28d-cured specimens. In addition to the environment and the conditioning regimes (stray current or anodic polarization), the following factors affect the observed behavior: (1) the steel surface properties prior to conditioning; (2) the properties of the mortar bulk matrix, such as the maturity and pH of the pore solution; (3) the porosity and pore network connectivity of the mortar bulk determining ion migration, as well as water transport and oxygen penetration.

The steel surface property is a factor that is relevant for both 24h and 28d-cured groups. This factor is important in the sense that a clean steel surface would be relatively more active compared to oxide layer-covered (“as received”) steel. This factor will dominate until a stable passive layer is formed (as in the non-corroding specimens) in the high pH environment of the pore solution in mortar.

The effects linked to changes in cement-based material properties, together with their influence on passive film formation, would be more significant in the 24h-cured group. If the stray current supply (or anodic polarization) starts at very early age (e.g., at the age of 24h), water transport and leaching-out effects, due to ion migration, will be more evident, because of the high porosity and pore network connectivity of a fresh bulk. Hence, if the hydration process and pore network characteristics are influenced by the foregoing factors, these will further affect the stability of the passive and/or corrosion product layer.

#### 3.1.1. OCP and R_p_ of 24h-Cured Specimens

As can be observed in Figure 3, until 28 days of age, the majority of OCP values for specimens R-24h (reference case) and S-24h (stray current case) fall in the cathodic region and are far beyond the passivity threshold, i.e., −200 ± 70 mV (vs. SCE) for reinforced mortar/concrete systems. These OCP values reflect the active state of the steel for at least the first 28 days of age. This is because of the clean surface of the embedded steel and the fresh mortar bulk. Specifically, the electrochemical cleaning of the embedded steel performed prior to casting results in a “bare” steel surface, which will be active in an alkaline environment of pH > 13.5 at a very early age, until a passive layer is formed and the pH of the environment (pore solution) stabilizes at around 12.9.

As can be observed in Figure 4, relatively low R_p_ values are initially recorded for the reference specimen R-24h, and they increase to 60 kΩ∙cm^2^ finally. This was not as expected, although it is in line with the OCP evolution for R-24 in Figure 4. Until 141 days, higher R_p_ values (in the range of 40–70 kΩ∙cm^2^) for S-24h (compared to R-24h) are recorded. For specimen A-24h, very low R_p_ values are recorded, below 10 kΩ∙cm^2^, with an increasing trend toward 30–40 kΩ∙cm^2^ between 14d and 141d. A significant reduction of R_p_ values for A-24h, below 5 kΩ∙cm^2^, is observed at the end of the test (at 215 days).

In the period of 3 days until 28 days for the 24h-cured groups, the OCP records reflect the steel electrochemical response within a gradually refined pore network: a steel–cement pate interface development, which is characteristic for a cement-based system at the early hydration stage. These are in terms of pore solution chemistry alterations as well as passive layer stabilization (for reference cases) or corrosion initiation (for corroding cases). The development of the passive layer and further stabilization is illustrated by the initial fluctuations of OCP values for the reference group R-24h and stabilization further on, toward more anodic OCP values and higher R_p_ values after prolonged conditioning (see Figure 3, after the age of 215 days).

A factor related to the above observations, which contributes to an impeded passive layer formation, is the leaching-out effect. If a cement-based material is in a prolonged contact with water, the dissolution of cement hydrates will occur (due to alkali ions—Ca^2+^, Na^+^, K^+^ leaching) [13,14,15]. The transport of sodium and potassium is faster than that of calcium ions, and it is more pronounced at early stages (a fresh bulk matrix means a non-mature, more open pore structure), whereas it is stable and/or negligible with longer treatment [16,17]. The leaching of calcium ions promotes coarsening of the pore structure, and it leads to increased transport properties (permeability, diffusivity) and a decrease in the mechanical properties [17].

Generally, the leaching process starts with a total dissolution of portlandite (calcium hydroxide, CH), ettringite, followed by a progressive decalcification of the calcium–silicate–hydrate (C-S-H) phase [18,19]. From 1 day of age onwards (after 24 h curing in molds), the 24h-cured specimen (R-24h) was conditioned in water, which is likely to result in leaching-out and altered transport properties of the bulk matrix. Hence, stabilization of the passive layer in a fresh (24h only cured) cement-based system, as in specimen R-24h, takes a significantly longer period—after 141 days of conditioning (see Figure 3), when OCPs tend toward more anodic values.

It can be noted that the recorded R_p_ values of C-24h are higher than that for the reference group R-24, especially in the first period of 3-28 days (Figure 4). This phenomenon is due to the effect of NaCl as an accelerator of cement hydration (especially at early age before 28 days) [20,21,22]. In case of NaCl additions, Friedel’s salt is formed, and it is accompanied by a release of NaOH, which is attributed to the chemical action between NaCl and 3CaO∙Al_2_O_3_∙6H_2_O. Consequently, the pH in the pore solution will increase. The increased pH will further accelerate the hydration process and modify the pore structure toward a finer one. This will lead to a more stable product layer on the steel surface, which in turn will delay Cl-induced corrosion damage. Therefore, initially higher R_p_ values are recorded for C-24h at an early age. After 28 days, the R_p_ values of C-24h remain at stable and lower values toward the end of the test, indicating the corroding state of C-24h after prolonged conditioning in 5% NaCl.

Until 141 days, the OCPs of specimens A-24h and S-24h are more noble than those for R-24h (in the range of −300 to −400 mV for S-24h and −300 to −100 mV for A-24h). A significant cathodic drop in the OCP value i.e., increased corrosion activity, for specimen A-24h is observed at the end of the test, establishing an OCP at around −600 mV. The OCP values for specimens S-24h remain stable over most of the test duration, with a cathodic shift and stabilization (around −400 mV) at the end of the test.

The stray current in the Cl-free condition (group S-24h, cured for only 24h) was expected to have a negative effect on steel corrosion resistance at an early age. However, this is not observed. On the contrary, the recorded OCP values (as shown in Figure 3) of specimen S-24h are more anodic than those for specimen R-24h (before 141d) and maintain stability (corresponding to higher R_p_ values of S-24h than those of R-24h, before 141 days, see Figure 4), suggesting an intensified process of passive layer formation/stabilization of S-24h at an early age. The more anodic OCP of S-24h can also be due to microstructural changes (e.g., a denser bulk matrix) at an early age. This will lead to an improved steel–mortar interface and a more stable passive layer.

Again, this observation reflects the effect of a “fresh” matrix on the properties of the steel–mortar interface. For group S-24h, stray current flow through the fresh (non-mature) cement matrix leads to enhanced water and ion transport due to a potential gradient. Hence, the cement hydration would be enhanced, a faster development of the cementitious microstructure would be at hand, and the more rapid stabilization of the pore solution and hydration products would occur at the steel–mortar interface. Previously reported and known are the early stage beneficial effects of a stray current on cement-based matrix properties [23].

#### 3.1.2. OCP and R_p_ of 28 Days-Cured Specimens

The outflow of stray current from the steel “body” accelerates corrosion on the steel surface (as shown in Figure 1a). This is relevant for all tested series related to stray current (i.e., “S” specimens), irrespective of the curing duration (in fog room) prior to conditioning. However, the effect of stray current for the previously discussed S-24h specimen is already different for the S-28d specimen (cured for 28d). For S-28d, the stray current was applied when the bulk matrix was already hardened. In this situation, ion and water transport cannot be as significantly enhanced, as this would be in a fresh matrix (e.g., as in S-24h). Therefore, the effect of stray current on cement hydration of S-28d is slight, and the stray current effect would be mainly on the properties of the product layer on the steel surface of S-28d.

As can be observed in Figure 6, the R_p_ values of S-28d show a trend toward lower R_p_ (110 kΩ∙cm^2^, at 243 days), i.e., lower corrosion resistance, if compared to the 28d-cured reference specimen R-28d (590 kΩ∙cm^2^, at 243 days). In line with the R_p_ records and compared to the 28d-cured reference case (R-28d), more cathodic OCP values of ca. −290 mV are observed for group S-28d at the time interval of 243 days.

The R_p_ values of R-28d (Figure 6) are higher than that for R-24h (see Figure 4) over the testing period. These, together with the more noble OCP values of R-28d, show the more resistive steel surface of R-28d, and they reflect that a sufficient curing leads to a stable product layer formation on the steel surface in a Cl-free environment. The expected beneficial effect of sufficient curing is also reflected by the higher R_p_ values of A-28d specimen compared to those of A-24h.

The corroding specimens cured for 28d (C-28d, CS-28d, and CA-28d), exhibit cathodic OCP values at the end of conditioning. In accordance with these cathodic OCPs, the R_p_ values of C-28d and CS-28d are much lower than those of R-28d and S-28d. Lower R_p_ is recorded for CS-28d (25 kΩ∙cm² at 243 days), compared to C-28d (about 40 kΩ∙cm² at 243 days). The result illustrates the effect of both Cl-induced corrosion and the additional stray current contribution in the case of a CS specimen. The most active steel surface is observed for CA-28d (the lowest R_p_ values are recorded for CA-28d after 28 days).

Based on the OCP and LPR results, it can be concluded that stray current and anodic polarization exert significantly different effects on the corrosion behavior of steel embedded in mortar, in both 24h-cured (samples cured in a fog room for only 24h) and 28d-cured (samples cured in a fog room for the standard 28 days) conditions. This will be discussed in more detail together with EIS response in the next sections.

### 3.2. Curing Effect Reflected by EIS Response

For a reinforced cement-based system, qualification of the EIS is a useful approach for evaluating the corrosion state of steel and a simplified assessment of the electrical properties of the bulk matrix. For instance, a reinforced mortar specimen conditioned in NaCl will logically perform differently in time if compared to a reference specimen conditioned in water. This is due to the expected Cl-induced steel corrosion in the former case and stabilization of the passive state of the steel reinforcement in the latter case.

Additionally, alterations in the electrical properties of the cement-based matrix, e.g., increased resistivity over time, would be expected because of the cement hydration [24]. Similarly, factors such as chemical composition of the external environment, ion and water penetration into the bulk matrix, pore interconnectivity of bulk matrix, variation in bulk matrix diffusivity, etc., will determine changes in the electrical properties of the bulk matrix over time [20,25,26]. These can be reflected by the high to middle frequency of the EIS response. All these features in an experimental EIS response are well visible and can be compared qualitatively for systems as in this work—reference and corroding reinforced mortar specimens. The EIS responses at time intervals of the most significant interests will be presented and discussed in this work.

**Figure 7:**
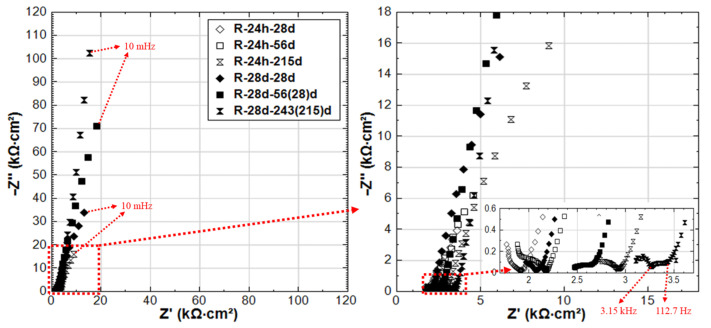
Electrochemical Impedance Spectroscopy (EIS) responses overlay in Nyquist format of R-24h and R-28d (R-24h: Reference, 24h-cured specimen treated in water; R-28d: Reference, 28d-cured specimen treated in water).

**Figure 8:**
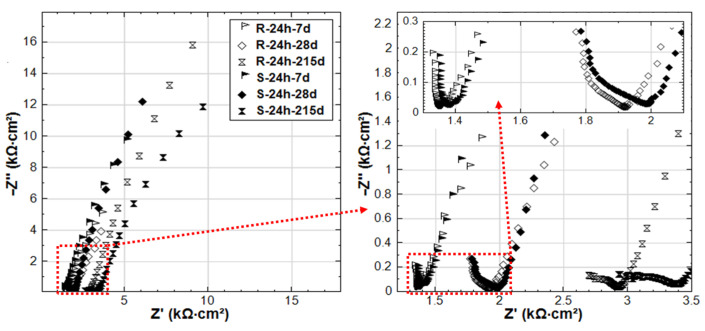
EIS responses overlay in Nyquist format of R-24h and S-24h (R-24h: Reference, S-24h: Stray Current, 24h-cured specimens treated in water).

The EIS responses overlay in Nyquist format of R-24h and R-28d are shown in Figure 7. The responses for 24h-cured groups (R-24h and S-24h) are presented in Figure 8. These cases are chosen here to address the effect of curing (Figure 7) on one hand. On the other hand, the comparison of R-24h and S-24h cases (Figure 8) would specifically address the aspect of the stray current effect.

For the 24h-cured reference group R-24h, the shape of the experimental curves reflects the typical response of steel in a Cl-free cement-based environment (alkaline medium) [27]. The response means EIS curves inclined to the y-axis, specifically in the LF EIS response range, denoting a capacitive-like behavior (passive state of the steel). This is relevant for both R-24h and R-28d at the end of conditioning, denoting the stabilization of the passive layer over time.

A stable passive state would be related to R-28d from the beginning of the test (28d response), but stability of the passive layer for the R-24h group would develop over time of conditioning, as can be observed in Figure 7 for the 215d response. Figure 7 also depicts variation in bulk matrix properties for the 24h and 28d-cured groups, as reflected by the HF EIS response (Figure 7, inlet). As can be observed, the magnitude of real |Z| increases over time (due to cement hydration in both cases), but it ends up higher for the 28d group at 215d of conditioning. For the last time intervals (after 215d conditioning), an additional time constant (in the range of 3.15 kHz–112.7 Hz, Figure 7 inlet) can be observed in the HF response of R-28d, while it is not observed for R-24h. This time constant of R-28d reflects a more developed bulk matrix, steel–mortar interface, and product layer on the steel surface. Evidently, a more corrosion resistive product layer is formed in R-28d, as also reflected by the LF response for this specimen (Figure 7). All these results show the importance of sufficient curing duration for the development of both bulk matrix properties and passive layer formation at the steel–mortar interface.

Related to ion transport in the bulk matrix, the main difference between R-24h and S-24h is the effect of ion and water migration (as in “S” cases) compared to diffusion controlled processes only (as in “R” cases). Ion migration is logically expected to affect both pore network properties and passive layer formation. As can be seen in Figure 8, the Z’ values in the HF EIS response (inlet in Figure 8) increase with the time of conditioning for both R-24h and S-24h, indicating the increase of bulk matrix resistance due to the ongoing cement hydration. For specimen S-24h, the magnitude of HF Z’ is slightly higher than that for R-24h at the initial time intervals of 7–28 days. In the meanwhile, the LF responses of S-24h (at 7 and 28 days) reflect a more corrosion resistant steel surface compared to R-24h. Both LF and HF responses before 28 days for specimens R-24h and S-24h are in line with the OCP and R_p_ values, where the more noble potential and higher R_p_ are recorded for S-24h. All these results suggest an intensified process of cement hydration and passive layer formation/stabilization of S-24h at earlier ages if compared to the reference condition (R-24h).

In contrast to the above performance, the responses of S-24h and R-24h change toward the end of the test, namely: the LF response for S-24h depicts a reduction of |Z|, which is well in line with the more cathodic OCP (Figure 3) and lower R_p_ (Figure 4) for the same time interval. The corrosion resistance of R-24 increases at the end of conditioning, which is reflected by the increasing |Z| in the LF (Figure 7), the more noble OCP (Figure 3), and an increasing trend of the R_p_ (Figure 4). All these mean that the stray current (negative) effect is observed but at already later stages for the group of 24h-cured specimens.

**Figure 9:**
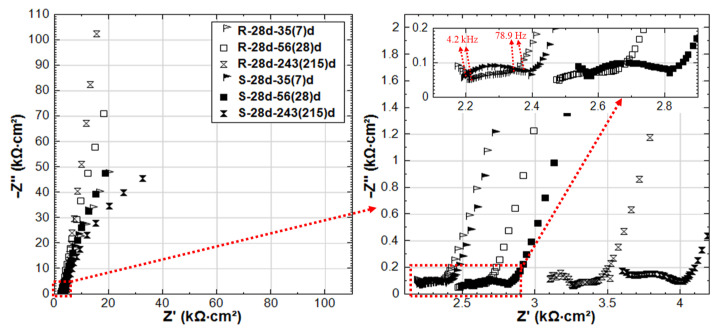
EIS responses overlay in Nyquist format of R-28d and S-28d (R-28d: Reference, S-28d: Stray Current, 28d-cured specimens treated in water).

The experimental impedance responses for the 28d-cured specimens R-28d and S-28d can be seen in Figure 9. With regard to group R-28d, the curves reflect the passivation of the steel reinforcement. It can be noted that the time constant (in the range of 4.2 kHz–78.9 Hz, as marked in inlet of Figure 9) is relevant for both R-28d and S-28d. In this frequency window, the time constant reflects bulk matrix characteristics, showing a potentially higher portion of the disconnected pore-network and hence a more resistive bulk matrix in both R-28d and S-28d compared to the previously discussed 24h-cured groups (R-24h and S-24h).

Similar to the 24h-cured group, the EIS response for the 28d group depicts a negative effect of the stray current, which is predominant at the later stages. Although an improvement of the bulk matrix is also observed here (Figure 9), the magnitude of |Z| in the LF for specimen S-28d is 2-time lower than that of R-28 at the end of the test. In other words, the effect of curing (group 28d vs group 24h) only results in a delay of the negative effect of stray current, rather than preventing it.

### 3.3. Competitive Mechanisms of Stray Current and Cl^−^ at Early Age

**Figure 10:**
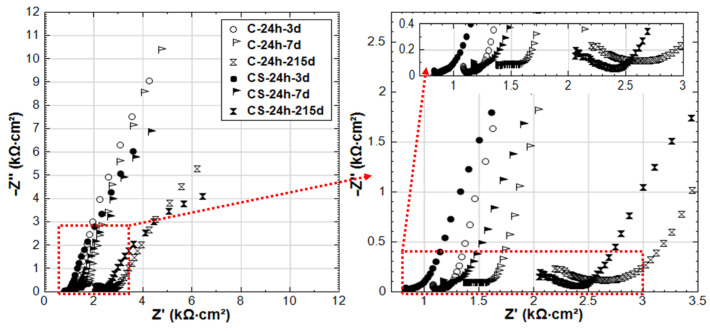
EIS responses overlay in Nyquist format of C-24h and CS-24h (C-24h: NaCl medium, CS-24h: NaCl + Stray Current, 24h-cured specimens treated in 5% NaCl).

**Figure 11:**
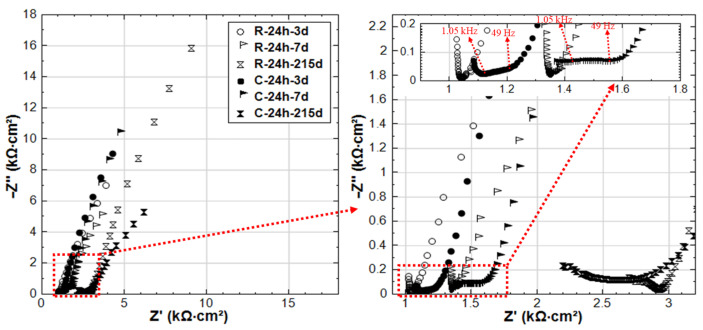
EIS responses overlay in Nyquist format of R-24h and C-24h (R-24h: Reference, 24h-cured specimens treated in water; C-24h: NaCl medium, 24h-cured specimens treated in 5% NaCl).

In contrast to groups R-24h and S-24h, the EIS responses of groups C-24h and CS-24h (specimens cured for 24 h, and then immersed in 5% NaCl solution) show clear evidence of active corrosion (see Figure 10). As already mentioned, the recorded R_p_ (derived from LPR) for C-24h is higher than that of R-24 at an early age (period of 3–28 days, see Figure 4). This is also reflected by the EIS as shown in Figure 11: the LF |Z| of C-24 (at intervals of 3d and 7d) is higher than that of R-24.

On the one hand, the arrival of Cl^−^ at the steel surface needs time, as the penetration of Cl^−^ in mortar cover is hysteretic. In this penetration process of Cl^−^, the acceleration of cement hydration is triggered in the meanwhile, resulting in a denser bulk matrix (chloride additions in ordinary Portland cement materials increase the volume of finer pores and decrease the fraction of coarse pores [21,22,28,29]). In turn, this denser bulk matrix hinders the further penetration of Cl^−^. The denser bulk matrix of C-24h compared to R-24h can be verified by the HF response in Figure 11: the |Z| values of C-24h of HF are higher than R-24 before 28 days, and an additional time constant (a depressed semi-circle) in the range of 1.05 kHz–49 Hz can be observed in the EIS responses of C-24h-3d and C-24h-7d. This response is typical for a reinforced cement-based system in the presence of Cl^−^, and it signifies the effect of Cl^−^ on bulk matrix properties (densification in this case).

An additional effect related to specimen C-24h is the potentially lower [Cl^−^]/[OH^−^] ratio at the steel surface. At a very early age in a fresh cement matrix, the OH^−^ concentration is relatively high; hence, together with the low concentration of Cl^−^ at the initial time intervals, a more stable product layer will form on the steel surface at an early age. However, after 28 days, the passive layer breakdown of C-24h is already evident. This is reflected by the low R_p_ values derived from LPR (see Figure 4) and by the EIS response for group C-24h, where a semi-circle inclined to an x-axis (real axis) and a decreasing |Z| toward 215 days are recorded. The shape of this EIS response had been largely reported to be due to the presence of Cl^−^ on the steel surface and the increasingly active corrosion state [30].

Comparing the EIS response of C-24h and CS-24h (see Figure 10), and also in line with the LPR results, a more active state is recorded for CS-24h, which is reflected by the LF of the EIS response at the end of conditioning. This is because of the synergetic effect of the stray current and Cl^−^; i.e., after prolonged conditioning of the stray current, the more anodic zones are produced where the current leaves the steel surface. This, together with the Cl^−^, leads to the lower corrosion resistance of CS-24h at the end of conditioning.

The more active state in CS-24h is accompanied by lower |Z| values in the HF domain for CS-24h compared to C-24h at all time intervals (Figure 10), meaning a lower resistance of the mortar bulk matrix for CS-24h. As already discussed, NaCl leads to cement hydration acceleration and results in densification of the matrix (higher resistance). Cl^−^ and Na^+^ ion migration is also accelerated by the potential gradient induced by stray current (as in CS-24h); hence, ions will migrate more easily, and the distribution of ions in the bulk matrix of CS-24h will be more uniform than that in the case without stray current (C-24h). Consequently, the Cl^−^ ions will more easily reach the steel surface when stray current is involved, especially for the “fresh” bulk matrix with high porosity and more connected pore networks at an early age.

**Figure 12:**
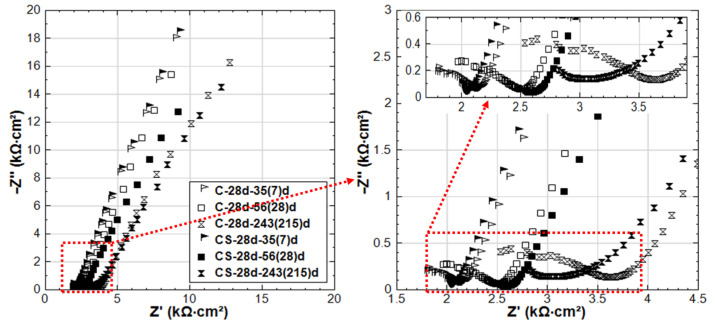
EIS responses overlay in Nyquist format of C-28d and CS-28d (C-28d: NaCl medium, CS-28d: NaCl + Stray Current, 28d-cured specimens treated in 5% NaCl).

As for C-28d and CS-28d (see Figure 12), much higher corrosion resistance compared to C-24h and CS-24h is observed. This reflects the curing effect: sufficient curing and hydration of the bulk matrix lead to a more resistive steel surface. At the age of 35 days (after 7 days of stray current supply), the EIS responses of C-28d and CS-28d are similar. In other words, the stray current effect is not significant yet at this stage. Even after 28 days of current supply, although the steel surface of CS-28d shows a slightly enhanced corrosion activity, the HF EIS responses of C-28d and CS-28d are still similar and in the same range of |Z| values. This is in contrast to the previously discussed CS-24h, where after only 3–7 days of stray current supply, the stray current already played roles in both affecting the “fresh” bulk matrix and steel surface (Figure 10). At the end of the test duration, the EIS response of CS-28d-243(215)d shows lower |Z| and is more inclined to a real axis response compared to that of C-28d-243(215)d. This indicates a higher corrosion activity in CS-28d after a prolonged supply of stray current if compared to C-28d specimen, where only Cl-induced corrosion plays a role.

According to above observations, a hypothesis about the stray current effect at early age can be proposed: the competitive mechanisms act in specimen CS-24h, where on the one hand, the stray current has positive effects on bulk matrix properties, similar to specimen S-24h, at early stages. On the other hand, stray current accelerates Cl^−^ ion migration toward the steel surface, leading to Cl-induced corrosion and an active state of steel. It is also reported that stray current can accelerate the decomposition of the C-S-H gel and a subsequent desorption of the physically adsorbed Cl^−^ by the C-S-H gel [31].

In the case of CS-24h, when the stray current was supplied at the age of 24h (1d) on the specimen immerged in 5% NaCl, stray current would immediately flow through the bulk matrix and polarize the steel (since steel is the low resistive path in the system). In contrast, Cl^−^ needs to firstly penetrate into the mortar cover before it reaches the steel surface. This means that the effects of stray current are immediate, influencing both the steel surface and the bulk matrix (i.e., accelerating cement hydration). Both of these lead to product layer formation on the steel surface (compounds as Fe_3_O_4_: FeO + Fe_2_O_3_, γ-FeOOH, or γ-Fe_2_O_3_ would form). This would be a positive effect of the stray current on the steel–mortar interface at an early age, which is in line with the OCP and LPR (R_p_) results, as already discussed in Section 3.1. The product layer would remain stable before Cl^−^ ions arrive at the steel surface and exceed the threshold value of Cl^−^ concentration toward de-passivation (a transformation from the passive layer to 3Fe(OH)_2_∙FeCl_2_ due to Cl^−^).

For specimen CS-28d, the penetration of Cl^−^ into the bulk matrix will be delayed due to the well cured mortar cover (higher resistance, lower porosity, and lower connectivity of the pore network of the bulk matrix). The denser matrix in CS-28d is evident from the higher HF |Z| (Figure 12) compared to that of CS-24h (Figure 10) at all time intervals. After curing of 28 days, a more resistive product layer (compared to CS-24h) has also been formed on the steel surface of CS-28d (seen by the LF response of EIS, where the LF |Z| values of CS-28d are higher than CS-24h). In this situation (CS-28d), the stray current effect is less significant on both the bulk matrix and the steel surface. Yet, after prolonged conditioning, the stray current still takes effect on the well-cured bulk matrix. As can be seen in Figure 12, the HF |Z| of CS-28d is lower than C-28d at age of 243 days. Compared to C-28d, the steel surface of CS-28d is more active at 243 days (see Figure 12, the LF response of EIS reflect this), indicating that the stray current accelerates steel corrosion after prolonged conditioning.

### 3.4. EIS Response Indicating Difference between Stray Current and Anodic Polarization

**Figure 13:**
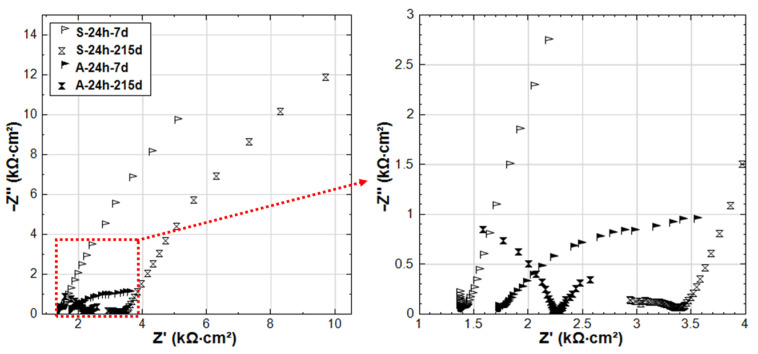
EIS responses overlay in Nyquist format of S-24h and A-24h (S-24h: Stray Current, A-24h: Anodic Polarization, 24h-cured specimens treated in water) at age of 7d and 215d.

**Figure 14:**
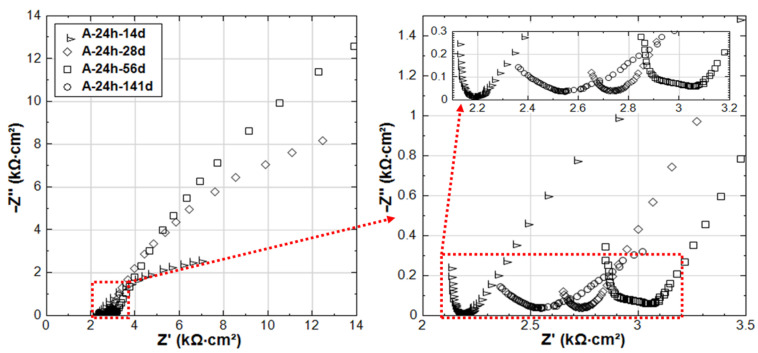
Overlay of EIS responses of A-24h in Nyquist format, at age of 14, 28, 56, and 141 days.

**Figure 15:**
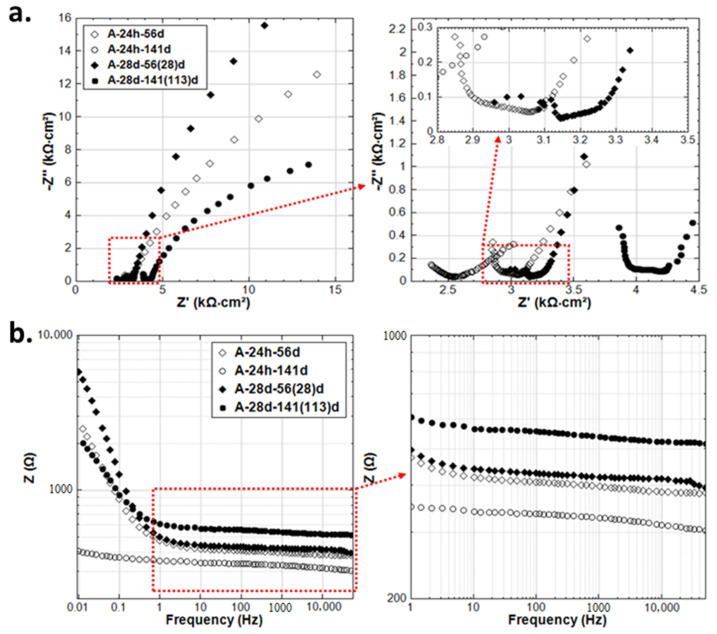
Overlay of EIS response of A-24h and A-28d in: (**a**) Nyquist format; (**b**) Bode format; at age of 56 and 141 days.

**Figure 16:**
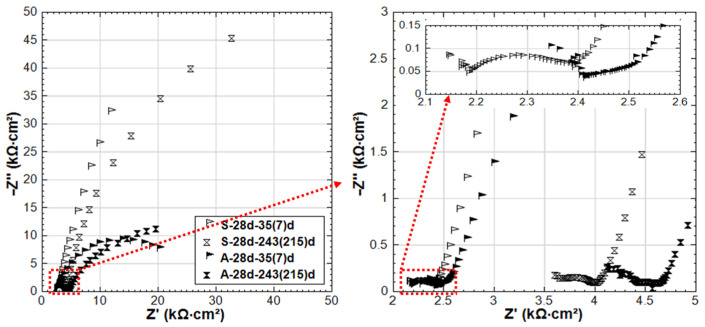
EIS responses overlay in Nyquist format of S-28d and A-28d (S-28d: Stray Current, A-28d: Anodic Polarization, 28d-cured specimens treated in water) at age of 35(7)d and 243(215)d.

**Figure 17:**
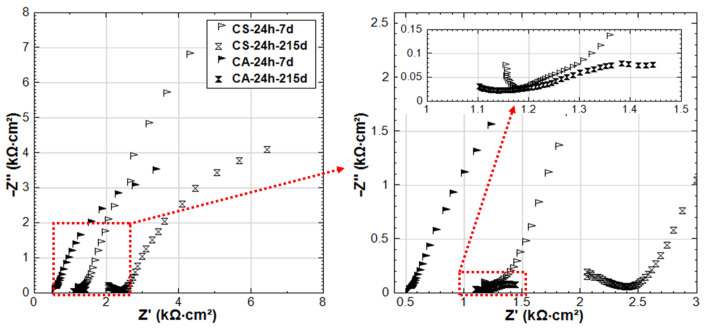
EIS responses overlay in Nyquist format of CS-24h and CA-24h (CS-24h: NaCl + Stray Current, CA-24h: NaCl + Anodic Polarization, 24h-cured specimens treated in 5% NaCl) at age of 7d and 215d.

**Figure 18:**
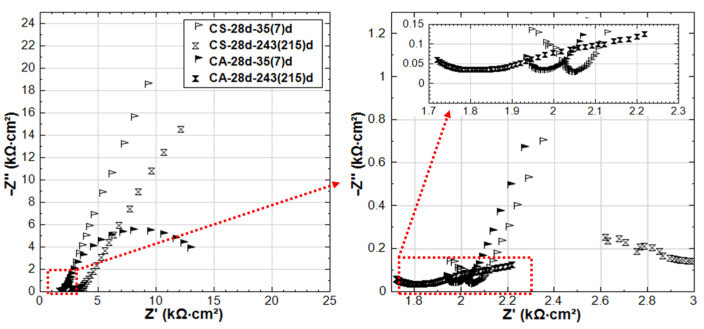
EIS responses overlay in Nyquist format of CS-28d and CA-28d (CS-28d: NaCl + Stray Current, CA-28d: NaCl + Anodic Polarization, 28d-cured specimens treated in 5% NaCl) at age of 35(7)d and 243(215)d.

The aim of this section is to clarify the different effects of stray current and anodic polarization, on specimens after 24h curing (fresh bulk matrix) or 28d curing (already hardened bulk matrix), in Cl-free or Cl-containing environments. To illustrate the different behavior of the rebar (by the EIS LF response) and mortar cover (by the EIS HF range) induced by stray current and anodic polarization, the overlays of relevant EIS response are shown in Figure 13, Figure 14, Figure 15, Figure 16, Figure 17, Figure 18. The comparison of EIS responses of S-24h and A-24h is shown in Figure 13. For the response of specimen A-24h, an actively corroding steel surface is already seen at 7 days of age. This behavior is in line with the cathodic OCP values for group A-24h (Figure 3) at the initial stage of the test (approximately −590 mV at 3 days, −320 mV at 7 days), as well as with the LPR-derived R_p_ values (Figure 4) of A-24h.

**Figure A1:**
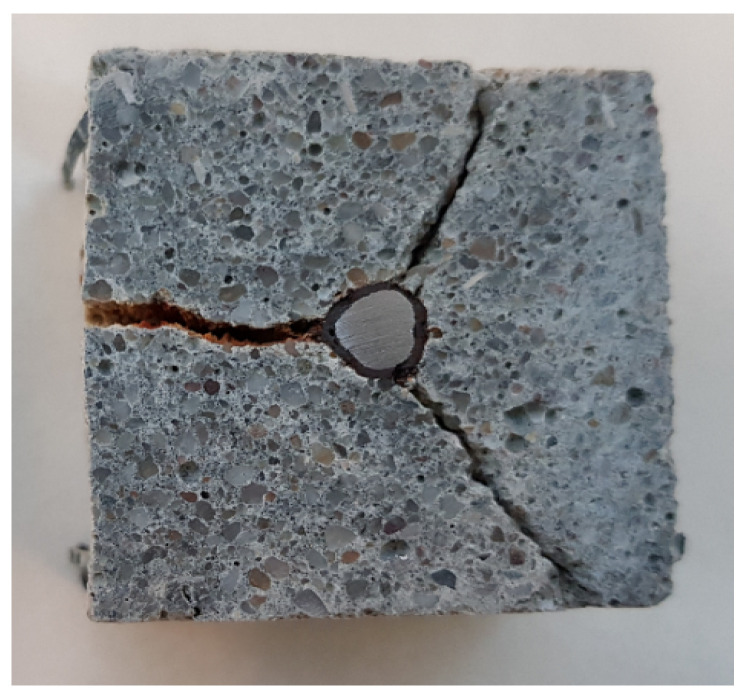
Cross-section of A-24h at the end of conditioning.

The more noble OCP values (Figure 3) recorded for A-24h before 56 days of age, together with the increased R_p_ before 141 days (Figure 4), are probably due to the specific product layer formation and compaction in conditions of anodic polarization [32,33]. This is also supported by the EIS response of A-24h (see Figure 14): after the active state at 14 days, as seen from the LF response, an enhanced corrosion resistance is observed at the stages of 28 and 56 days (increasing of |Z| with prolonged conditioning). It is well-known that with anodic polarization, in the absence of corrodents (as Cl^−^) and in alkaline medium (pH of 12.5–12.9, for concrete pore solution), a product layer will be forced to form (according to thermodynamical principles as reflected by the Pourbaix diagram of the Fe-H_2_O system [32]), i.e., with constant anodic current, the electrode potential will gradually rise toward a stable value [34,35,36]. A transformation to a more active state, together with a drop in bulk matrix resistance, is recorded later on after the stage of 141 days. This is ascribed to the anodic polarization-induced crack, by expansion of the corrosion product layer on the steel surface of A-24h (see Figure A1 in Appendix A). The reduction of R_p_ for A-24h (see Figure 4), together with a significant cathodic drop of OCP (Figure 3), accounts for an active state of A-24h at the end of the test. In contrast, such a significant corrosion activity is not observed for S-24h.

The above behavior is more significant for the 24h curing situation, i.e., A-24h, compared to A-28d (see Figure 15). As shown in Figure 15a, a clear difference in the HF and LF of A-24h and A-28d can be observed in all time intervals. This can be attributed to the sufficient and timely OH^−^ supply of A-24h, because of hydration process of cement at an early age. In the “fresh” matrix, the migration of OH^−^ (toward the steel surface) affected by the electrical field is enhanced. In the meanwhile, the Fe^2+^ is continuously produced by the supplied anodic current; consequently, more corrosion products are formed at the steel–mortar interface. The above processes are not significant for the already hardened bulk matrix of A-28d at later age.

As for A-28d, the evident semi-circle of EIS plots (Figure 16) is already recorded at 35 days of age (after 7 days conditioning). The EIS response in the LF domain inclines to the x-axis, indicating an active steel surface. This response again confirms that anodic polarization induces corrosion for reinforcing steel. Comparing the EIS response of S-28d and A-28d (standard curing for 28 days), a more significant corrosion behavior is recorded for A-28d. This is supported by the OCP and R_p_ (from LPR) values, as shown in Figure 5 and Figure 6. A capacitive ark (in MF range) is observed for A-28d at 215 days, meaning a potentially improved resistance of the interface, which can also be reflected by the higher |Z| of A-28d at 215d. This may be attributed to the expansion of the corrosion product at the steel–mortar interface and an altered steel–mortar interface of A-28d. More details will be further discussed together with potential decay recording in Section 3.5.

For the response of the CA groups (NaCl + anodic polarization), CA-24h shows a more significant corrosion activity than CS-24h (NaCl + stray current). At 215 days, a significant drop of impedance and phase angle is recorded for CA-24h (Figure 17). This response indicates the synergetic action of two degradation factors, i.e., anodic polarization and Cl^−^ in the environment, resulting in significantly low corrosion resistance. For this CA-24h case, the EIS response is well supported by the recorded cathodic OCP values and extremely low R_p_ values (Figure 3 and Figure 4).

A more active behavior is recorded for group CA-24h compared to CA-28d. At the end of conditioning, the EIS response of CA-28d shows a more significant corrosion of steel than A-28d and CS-28d. This again implies the coupling effects of anodic polarization and Cl^−^ on the accelerating corrosion of steel.

**Figure A2:**
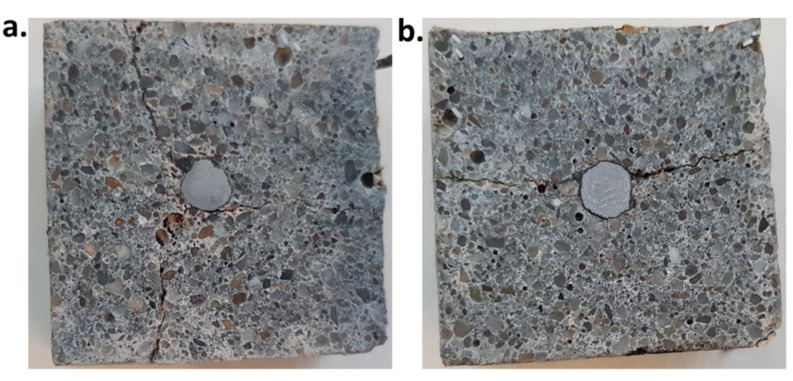
Cross-section of (**a**) CA-24h; (**b**) CA-28d at the end of conditioning.

For both 24h curing and 28d curing conditions, a more pronounced corrosion is observed for the CA groups (NaCl + anodic polarization, specimens CA-24h, and CA-28d) compared to the stray current groups (NaCl + stray current, CS-24h, and CS-28d). Visible/macro cracks induced by corrosion are observed for both CA-24h and CA-28d (see Figure A2, Appendix A). Once cracks are induced, the bulk matrix resistance (in 5% NaCl solution) will decrease, as the water and ions will penetrate into the bulk matrix via cracks. In a Cl-containing alkaline environment, a stable passive layer cannot form on the steel surface, although the anodic current was applied continuously. In this situation, Cl-containing iron oxides and hydroxides form on the steel surface, because of Cl^−^ ingression [30]. In addition, the anodic current applied to the steel rebar accelerates Cl^−^ ion migration toward the steel–mortar interface (as illustrated in Figure 1b). This synergy of anodic polarization and Cl^−^ significantly accelerates the corrosion propagation. In contrast, the synergy of stray current and Cl^−^ causes different effects (Figure 1a). Stray current leads to the formation of anodic zones (corroding areas) where the current leaves the steel surface. In other words, only part of the steel surface can be corroded by the stray current, while in conditions of anodic polarization, the whole steel surface acts as an anode.

Overall, stray current and anodic polarization exert significantly different effects on the corrosion behavior of steel and the surrounding mortar matrix in both 24h-cured and 28d-cured specimens. Regardless of whether there is Cl^−^ in the external environments, anodic polarization leads to more pronounced effects on corrosion behavior than stray current. Hence, anodic polarization cannot reflect the effects of stray current and should not be adopted to simulate stray current.

### 3.5. Potential Decay Monitoring over 24 Hours

Stray current leads to both cathodic and anodic polarization on the steel surface. The shift of the overall (mixed) potential induced by stray current flow can be adopted as an indicator of the presence of stray current. As aforementioned, for the specimens undergoing stray current or anodic polarization, a 24-hour potential decay measurement was performed and recorded prior to any further electrochemical tests. The reason for the decay tests is to verify:

(1) if the steel rebars were indeed affected by stray current (or anodic polarization, respectively); in other words, a change of potential in “ON” and “OFF” conditions, compared to rest conditions, will indicate polarization of the reinforcement due to current application;

(2) if an OCP stability would be achieved in rest conditions (when no external electrical field was applied), in the required time for the polarized system. This waiting time reflects the limitation of ion transport, diffusion, or limitation of electron transport along the steel surface, which are also related to the amount/thickness, heterogeneity, and composition of the product layer on the steel surface.

**Figure 19:**
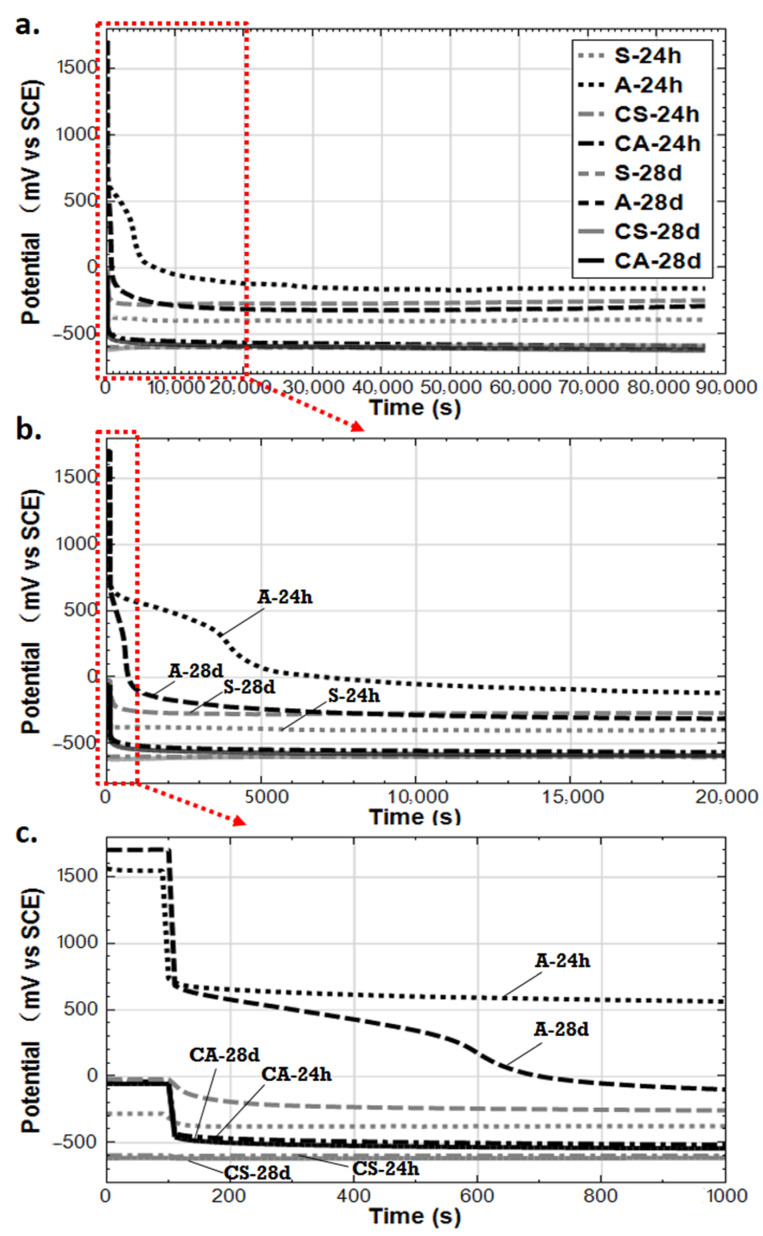
Potential decay monitoring over 24h de-polarization process (S-24h: Stray Current; CS-24h: NaCl + Stray Current; A-24h: Anodic Polarization; CA-24h: NaCl+ Anodic Polarization—After 24h curing; S-28d: Stray Current; CS-28d: NaCl + Stray Current; A-28d: Anodic Polarization; CA-28d: NaCl + Anodic Polarization—After 28d curing). (**a**) Full time scale; (**b**) Time scale of 0–20,000 s; (**c**) Time scale of 0–1000 s.

Figure 19 depicts the 24h potential decay results at the hydration age of 95 days. The very first recorded values (at the start of the curves and in the range of first 100 s) are the “ON-potentials”, as adopted due to anodic polarization or stray current, respectively (Figure 19c). At the moment of switching off the current supply, the potential drop reflects the contribution of the so-called “IR drop”. After a certain decay and toward the 24h time interval, the potentials of all specimens are stable (see Figure 19a).

The IR drop is the product of current (I) passing through resistance (with value of R) between the working electrode (the steel rebar) and the reference electrode (SCE). The “R” value is governed by the resistivity of the mortar matrix and the electrolyte (water or 5% NaCl in this case) surrounding the working electrode. The former is more significant and will depend on bulk matrix properties; hence, it will be different for the 24h and 28d-cured specimens. The latter (electrolyte resistance) is negligible in this set-up. After the IR drop, the potential decay reflects the relaxation of the system from the previous state of being under stray current or anodic polarization (“ON” conditions) toward the stability in “rest” conditions (“OFF” conditions).

As can be seen in Figure 19c, the IR drop for the anodic polarization cases (A-24h and A-28d) are the most pronounced. A higher IR drop is recorded for A-28d compared to A-24h (the IR drops for A-24h and A-28d are about 800 mV and 1000 mV, respectively). The higher IR drop for A-28d reflects the higher resistance of the bulk matrix compared with A-24h. The higher resistivity of the cement-based matrix partly indicates the higher hydration degree (maturity of concrete matrix) and denser bulk, which is as expected for specimen A-28d. This is in accordance with the EIS response at the HF domain of A-24h and A-28d. As shown in Figure 15, the real component of impedance Z’ in the HF range, reflecting the resistance of the bulk matrix, is lower for A-24h compared to that of A-28d at both 56 and 141 days.

For A-24h and A-28d, it is clear that longer time is needed for establishing an equilibrium condition at the steel-mortar interface of A-24h and A-28d (Figure 19b). This reflects the limitations of ion transport at the steel-mortar interface. In other words, at the age of 95 days, a stable steel surface in this environment (mortar pore solution without Cl^−^, alkaline medium, pH of 12.5–12.9) was formed due to the anodic polarization. It can be seen that the “instant-off potentials” of A-24h and A-28d are about 700 mV (vs. SCE). These “instant-off potentials” are the potential of steel after the disappearance of IR drop (i.e., instantaneously after the cutting-off of the current). In an alkaline environment and at anodic potentials higher than +300 mV (vs. SCE), a passive film formed at the steel surface [33]. By the anodic polarization of iron, Fe(III) may be formed directly on the electrode surface as Fe_2_O_3_, as mixed iron oxide Fe_3_O_4_, or as FeOOH [37,38,39,40,41,42].

A longer time is needed for establishing an equilibrium condition at the steel-mortar interface of A-24h compared to A-28d. This means that a thicker corrosion product layer was formed in A-24h. As aforementioned, this is attributed to the enrichment of Fe^2+^ produced by anodic polarization at a very early age (just at age of 1 day) at the steel-mortar interface. In the meanwhile, sufficient and timely OH^−^ supply in a “fresh” matrix at early age enhances the migration of OH^−^ to the steel surface in condition of the present electrical field. After this, the continuous supply of anodic polarization leads to the higher Fe^3+^/Fe^2+^ ratio in the passive film, which further enhances the stability of the product layer on the steel surface in A-24h until the age of 95 days when the potential decay was performed. This is in line with the OCP values and R_p_ (derived from LPR) of A-24h: noble OCP values are recorded for A-24h before 56 days of age, together with the increasing trend of R_p_ before 141 days, showing the product layer formation and stabilization process.

For S-24h and S-28d undergoing stray current interference, “instant-off potential” values are much lower (−300 mV for S-24h, 0 mV for S-28d) than those of A-24h and A-28d. Only around 100 mV of IR drop is monitored for S-24h and S-28d. Considering that the resistance (R component of IR) between the steel (working electrode) and reference electrode (SCE) is similar, the IR drop difference is mainly attributed to the difference of “I” (current flowing from the steel surface to the reference electrode). The much lower IR drop of S-24h and S-28d denotes the significantly lower current level flowing into the steel, comparing to the anodic polarization cases (A-24h and A-28d).

An IR drop higher than 400 mV is recorded for CA-24h and CA-28d. However, for CS-24h and CA-28d, no evident IR drop can be identified. It can also be found that the IR drop in Cl-containing cases is much lower than that of Cl-free specimens. This is due to the reduced resistance in the external environment of 5% NaCl solution. All these results again denote the different effects of stray current and anodic polarization in both Cl-free and Cl-containing environments.

## 4. Conclusions

In this work, the effects of stray current on the corrosion of reinforcing steel embedded in mortar are studied in view of electrochemical behavior in both 24h-curing and 28d-curing regimes. The comparison between stray current and anodic polarization is conducted in Cl-free and Cl-containing conditions. From the experimental results, the following conclusions can be drawn:

1. Stray current and anodic polarization exert significantly different effects on the corrosion behavior of steel embedded in mortar, in both 24h curing (the samples are cured in a fog room for only 24h) and 28d curing (the samples are cured in a fog room for 28 days) regimes. Anodic polarization simulating stray current means the absence of cathodic polarization and different corrosion mechanisms. Consequently, anodic polarization cannot reflect the effects of stray current, and therefore, it has limited significance for simulating stray current.
2. The curing regimes and starting time of stray current (i.e., the stray current supply starts at age of 24h or 28d) play significant roles in the formation of a corrosion product layer on the steel surface. At a very early age, water transport, leaching-out effects, and ion migration governed by the electrical field are more evident because of the high porosity and pore network connectivity of a fresh bulk. The hydration process and pore network characteristics are influenced by the foregoing factors, and they further affect the stability of the corrosion product layer.
3. In a Cl-free (conditioned in water) situation, stray current flowing through the fresh (non-mature) bulk matrix may lead to the enhanced migration of water and ions. In this case, the cement hydration and steel surface passivation can be enhanced. However, this phenomenon is not evident for the condition of sufficiently cured bulk matrix, as the bulk matrix is already hardened when the stray current is supplied. This also means that the properties of the cementitious material in a reinforced cement-based system are of significant importance and largely determine the electrochemical state of the steel rebar.
4. At an early age, competitive mechanisms act in specimen CS-24h (Cl^−^ + stray current, cured in fog room for only 24h). On the one hand, the stray current has positive effects: stray current flow through a fresh (non-mature) cement matrix leads to enhanced water and ion transport due to migration. The results are enhanced cement hydration and consequently a more rapid stabilization of pore solution and the steel–mortar interface. On the other hand, stray current enhances Cl^−^ ion migration and accelerates Cl-induced corrosion.

## Figures and Tables

**Table 1:** Summary of curing and conditioning regimes.

Group	Curing	Immersion Environment		Electrical Field	
		Water	5% NaCl	Stray Current	Anodic Polarization
R-24h	24h	√			
C-24h			√		
S-24h		√		√	
CS-24h			√	√	
A-24h		√			√
CA-24h			√		√
R-28d	28d	√			
C-28d			√		
S-28d		√		√	
CS-28d			√	√	
A-28d		√			√
CA-28d			√		√

R-24h: Reference; C-24h: Corroding (NaCl medium); S-24h: Stray Current; CS-24h: Corroding (NaCl) + Stray Current; A-24h: Anodic Polarization; CA-24h: Corroding (NaCl) + Anodic Polarization—After 24h curing; R-28d: Reference; C-28d: Corroding (NaCl medium); S-28d: Stray Current; CS-28d: Corroding (NaCl) + Stray Current; A-28d: Anodic Polarization; CA-28d: Corroding (NaCl) + Anodic Polarization—After 28d curing.

## Data Availability

Data sharing not applicable.

## References

[1] Bertolini L., Carsana M., Pedeferri P. (2007). Corrosion behaviour of steel in concrete in the presence of stray current. Corros. Sci..

[2] Chen Z., Koleva D.A., van Breugel K., Miron R.D.L.E., Koleva D.A. (2017). Electrochemical tests in reinforced mortar undergoing stray current-induced corrosion. Concrete Durability: Cementitious Materials and Reinforced Concrete Properties, Behavior and Corrosion Resistance.

[3] Chen Z., Koleva D., van Breugel K. (2017). A review on stray current-induced steel corrosion in infrastructure. Corros. Rev..

[4] Hornbostel K., Larsen C.K., Geiker M.R. (2013). Relationship between concrete resistivity and corrosion rate—A literature review. Cem. Concr. Compos..

[5] García A., Castro-Fresno D., Polanco J.A. (2008). Evolution of penetration resistance in fresh concrete. Cem. Concr. Res..

[6] ASTM G1 (2003). Standard practice for preparing, cleaning, and evaluating corrosion test specimens. Am. Soc. Test. Mater..

[7] Alonso C., Castellote M., Andrade C. (2002). Chloride threshold dependence of pitting potential of reinforcements. Electrochim. Acta.

[8] Rengaswamy N.S., Srinivasan S., Balasubramanian T.M., Mahadeva Y.I., Nayak N.U., Bapu R.H.S. (1988). Corrosion survey of reinforced and prestressed concrete structures—Methodology of approach. Trans. SAEST.

[9] Andrade C., Alonso C. (1996). Corrosion rate monitoring in the laboratory and on-site. Constr. Build. Mater..

[10] González J.A., Molina A., Escudero M.L., Andrade C. (1985). Errors in the electrochemical evaluation of very small corrosion rates—I. polarization resistance method applied to corrosion of steel in concrete. Corros. Sci..

[11] Stern M., Geary A.L. (1957). Electrochemical polarization: I. A theoretical analysis of the shape of polarization curves. J. Electrochem. Soc..

[12] Wenger F., Galland J. (1990). Analysis of local corrosion of large metallic structures or reinforced concrete structures by electrochemical impedance spectroscopy (EIS). Electrochim. Acta.

[13] Leemann A., Lothenbach B. (2008). The influence of potassium-sodium ratio in cement on concrete expansion due to alkali-aggregate reaction. Cem. Concr. Res..

[14] Ishii K., Seki H., Fukute T., Ikawa K. (1998). Cathodic protection for prestressed concrete structures. Constr. Build. Mater..

[15] Chang J.J. (2002). A study of the bond degradation of rebar due to cathodic protection current. Cem. Concr. Res..

[16] Saito H., Nakane S., Ikari S., Fujiwara A. (1992). Preliminary experimental study on the deterioration of cementitious materials by an acceleration method. Nucl. Eng. Des..

[17] Susanto A., Koleva D.A., Van Breugel K., Van Beek K. (2017). Stray current-induced development of cement-based microstructure in water-submerged, Ca(OH)_2_-submerged and sealed conditions. J. Adv. Concr. Technol..

[18] Ulm F.J., Torrenti J.M., Adenot F. (1999). Chemoporoplasticity of calcium leaching in concrete. J. Eng. Mech..

[19] Kuhl D., Bangert F., Meschke G. (2004). Coupled chemo-mechanical deterioration of cementitious materials. Part I: Modeling. Int. J. Solids Struct..

[20] Koleva D.A., Hu J., Fraaij A.L.A., van Breugel K., de Wit J.H.W. (2007). Microstructural analysis of plain and reinforced mortars under chloride-induced deterioration. Cem. Concr. Res..

[21] Haque M.N., Kayyali O.A. (1995). Free and water soluble chloride in concrete. Cem. Concr. Res..

[22] Pruckner F., Gjørv O.E. (2004). Effect of CaCl_2_ and NaCl additions on concrete corrosivity. Cem. Concr. Res..

[23] Susanto A., Koleva D.A., Copuroglu O., van Beek K., van Breugel K. (2013). Mechanical electrical and microstructural properties of cement-based materials in conditions of stray current flow. J. Adv. Concr. Technol..

[24] Siegwart M., Lyness J.F., McFarland B.J. (2003). Change of pore size in concrete due to electrochemical chloride extraction and possible implications for the migration of ions. Cem. Concr. Res..

[25] Koleva D.A., Copuroglu O., van Breugel K., Ye G., de Wit J.H.W. (2008). Electrical resistivity and microstructural properties of concrete materials in conditions of current flow. Cem. Concr. Compos..

[26] Koleva D.A., de Wit J.H.W., van Breugel K., Veleva L.P., van Westing E., Copuroglu O., Fraaij A.L.A. (2008). Correlation of microstructure, electrical properties and electrochemical phenomena in reinforced mortar. Breakdown to multi-phase interface structures. Part II: Pore network, electrical properties and electrochemical response. Mater. Charact..

[27] Keddam M., Nóvoa X.R., Soler L., Andrade C., Takenouti H. (1994). An equivalent electrical circuit of macrocell activity in facing electrodes embedded in cement mortar. Corros. Sci..

[28] Suryavanshi A.K., Scantlebury J.D., Lyon S.B. (1995). Pore size distribution of OPC & SRPC mortars in presence of chlorides. Cem. Concr. Res..

[29] Díaz B., Nóvoa X.R., Pérez M.C. (2006). Study of the chloride diffusion in mortar: A new method of determining diffusion coefficients based on impedance measurements. Cem. Concr. Compos..

[30] Koleva D.A., van Breugel K., de Wit J.H.W., van Westing E., Boshkov N., Fraaij A.L.A. (2007). Electrochemical behavior, microstructural analysis, and morphological observations in reinforced mortar subjected to chloride ingress. J. Electrochem. Soc..

[31] Chu H., Wang T., Guo M.-Z., Zhu Z., Jiang L., Pan C., Liu T. (2019). Effect of stray current on stability of bound chlorides in chloride and sulfate coexistence environment. Constr. Build. Mater..

[32] Pourbaix M. (1974). Atlas of Electrochemical Equilibria in Aqueous Solutions.

[33] Čekerevac M., Simičić M., Bujanović L.N., Popović N. (2012). The influence of silicate and sulphate anions on the anodic corrosion and the transpassivity of iron and silicon-rich steel in concentrated KOH solution. Corros. Sci..

[34] Takahashi K., Bardwell J.A., MacDougall B., Graham M.J. (1992). Mechanism of anodic dissolution and passivation of iron—II. Comparison of the behavior in neutral benzoate and acetate buffer solutions. Electrochim. Acta.

[35] Takahashi K., Bardwell J.A., MacDougall B., Graham M.J. (1992). Mechanism of anodic dissolution and passivation of iron—I. Behavior in neutral acetate buffer solutions. Electrochim. Acta.

[36] Ejaz A., Lu Z., Chen J., Xiao Q., Ru X., Han G., Shoji T. (2015). The effects of hydrogen on anodic dissolution and passivation of iron in alkaline solutions. Corros. Sci..

[37] Amaral S.T., Müller I.L. (1999). A RRDE study of the electrochemical behavior of iron in solutions containing silicate and sulphate at pH 10–13. Corros. Sci..

[38] Armstrong R.D., Baurhoo I. (1972). The dissolution of iron in concentrated alkali. J. Electroanal. Chem. Interfacial Electrochem..

[39] Burstein G.T., Askley G.W. (1983). Early steps in the anodic oxidation of iron in aqueous solution. Corrosion.

[40] Geana D., El Miligy A.A., Lorenz W.J. (1974). Electrochemical behaviour of iron in alkaline sulphate solutions. J. Appl. Electrochem..

[41] Schrebler Guzmán R.S., Vilche J.R., Arvía A.J. (1979). The potentiodynamic behaviour of iron in alkaline solutions. Electrochim. Acta.

[42] Suzuki S., Matsubara E., Komatsu T., Okamoto Y., Kanie K., Muramatsu A., Konishi H., Mizuki J., Waseda Y. (2007). Ex-situ and in-situ X-ray diffractions of corrosion products freshly formed on the surface of an iron-silicon alloy. Corros. Sci..

